# Precision diagnosis and therapy of a case of brain abscesses associated with asymptomatic pulmonary arteriovenous fistulas

**DOI:** 10.1186/s12879-020-05092-6

**Published:** 2020-05-24

**Authors:** Lu-Yao Gao, Guang-Run Xu, Ting-Jun Dai

**Affiliations:** 1 Department of Emergency Medicine and Chest Pain Center, Qilu Hospital of Shandong University, Jinan, 250012 Shandong Province China; 2 Department of Neurology, Qilu Hospital of Shandong University, Jinan, 250012 Shandong Province China

**Keywords:** Brain abscesses, Pulmonary arteriovenous fistulas, *Streptococcus intermedius*, Metagenomic next-generation sequencing

## Abstract

**Background:**

Brain abscesses, a severe infectious disease of the CNS, are usually caused by a variety of different pathogens, which include *Streptococcus intermedius (S. intermedius)*. Pulmonary arteriovenous fistulas (PAVFs), characterized by abnormal direct communication between pulmonary artery and vein, are a rare underlying cause of brain abscesses.

**Case presentation:**

The patient was a previous healthy 55-year-old man who presented with 5 days of headache and fever. Cerebral magnetic resonance imaging (MRI) suggested a brain abscess. Thoracic CT scan and angiography demonstrated PAVFs. Aiding by metagenomic next-generation sequencing (mNGS) of the cerebrospinal fluid (CSF) sample which identified *S. intermedius* as the causative pathogen, the patient was switched to the single therapy of large dose of penicillin G and was cured precisely and economically.

**Conclusions:**

It is an alternative way to perform mNGS to identify causative pathogens in patients with brain abscesses especially when the results of traditional bacterial culture were negative. Further thoracic CT or pulmonary angiography should also be undertaken to rule out PAVFs as the potential cause of brain abscess if the patient without any known premorbid history.

## Background

Brain abscesses are rare and life-threatening infectious diseases of the central nervous system (CNS) and can be caused by a variety of pathogens [1]. *Streptococcus intermedius* (*S. intermedius*) is a known pathogen that can cause brain abscesses [2]. Pathogens can spread directly to the brain from contiguous sinus infection, head trauma or neurosurgical procedures [3], or through a right-to-left shunt disease, such as patent foramen ovale [4], cyanotic cardiac disease [5] or pulmonary arteriovenous fistulas (PAVFs) [6]. PAVFs have abnormal direct communications between pulmonary artery and vein which called fistula [7]. Pathogens and embolus can enter the brain directly through fistula causing recurrent brain abscess or cerebral infarction. Reports of asymptomatic idiopathic PAVF-related brain abscesses are rare especially with identified pathogens. Herein we report a case of PAVF-related brain abscesses, in which the causative pathogen *S. intermedius* was detected and treated precisely aiding by metagenomic next-generation sequencing (mNGS).

## Case presentation

A 55-year-old man was admitted to the emergency room (ER) of Qilu Hospital, Shandong University on 12 October 2019. He had a 5-day history of fever (Max 39.0 °C) and headache, accompanied by chills, nausea, and vomiting, without epistaxis, cyanosis, exertional dyspnea or clubbing fingers. There was no history of head trauma, sinusitis, otitis media or dental infection. He also denied the history of hypertension, diabetes mellitus, lung disease or cardiovascular disease.

Upon admission, the patient had a slightly higher body temperature 37.2 °C, pulse rate was 88 beats/min, respiratory rate 21 breaths/min, blood pressure 105/60 mmHg. Physical examination revealed no neurologic deficit sign. Laboratory examination showed white blood cell (WBC) 10.99*10^9/L, neutrophil ratio (NEU%) 81.40%, lymphocyte ratio (LYM%) 12.5%, red blood cell (RBC) 4.55*10^12/L, hemoglobin 144 g/L, erythrocyte sedimentation rate (ESR) 43 mm/h, procalcitonin (PCT) 0.180 ng/ml (normal range < 0.1 ng/ml). Other blood tests, including blood glucose, sodium, alanine transaminase (ALT), aspartate aminotransferase (AST), the anti-nuclear antibodies (ANA), rheumatoid factor (RF), galactomannan enzyme immunoassay (GM-test), (1,3) beta-D-glucan assay (G-test)*,* T-SPOT, human immunodeficiency virus (HIV) were all within the normal range.

**Fig. 1:**
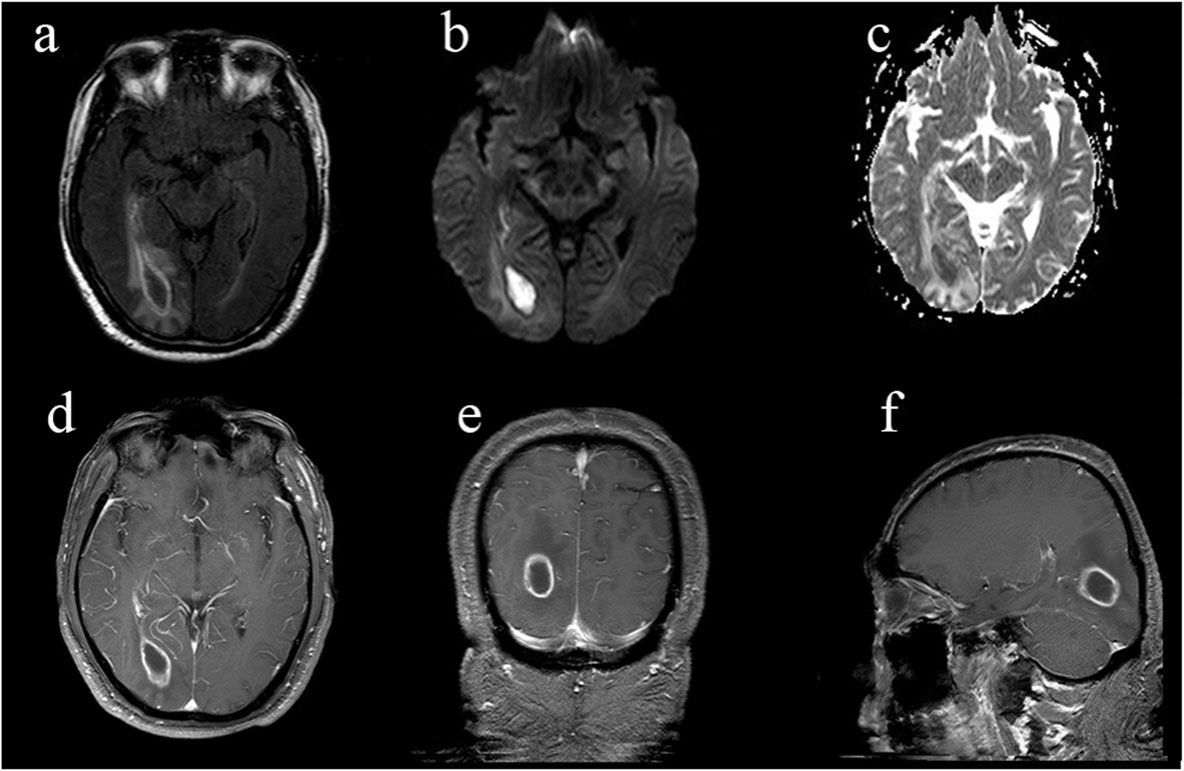
Brain MRI findings. **a** Axial FLAIR: a mass lesion with perilesional edema adjacent to right lateral ventricle occipital horn; the center of the lesion is hyperintense on DWI (**b**) and hypointense on ADC maps (**c**); after gadolinium injection, the lesion has a well-defined, thin-walled enhanced rim(**d**-**f**), which is consistent with the image findings of brain abscess. DWI, diffusion-weighted image; ADC, apparent diffusion coefficient

**Fig. 2:**
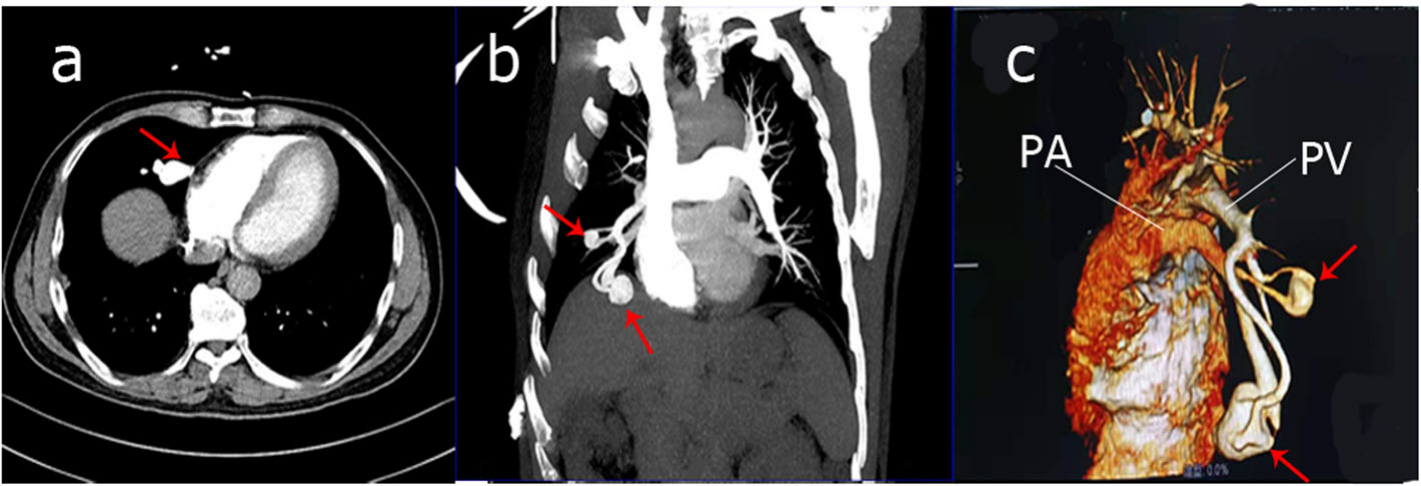
Pulmonary CTA findings. Axial thoracic CTA shows two irregular high-density nodules in the middle of right lung, maximum cross section 2.1 cm × 2.1 cm (**a**); (**b**) MIP and (**c**) VRT confirm PAVFs (red arrow). CTA, computed tomography angiography; MIP, maximum intensity projection; VRT, volumetric reproduction technique; PA, pulmonary artery; PV, pulmonary venous

Ultrasonic cardiogram showed no valvular vegetation. Brain enhanced magnetic resonance imaging (MRI) revealed a ring enhanced mass with perilesional edema adjacent to right lateral ventricle occipital horn (Fig. 1). Thoracic enhanced computed tomography (CT) displayed two irregular high-density nodules in the middle of the right lung, maximum cross section 2.1 cm*2.4 cm (Fig. 2a). Maximum intensity projection (MIP) and volumetric reproduction technique (VRT) of pulmonary CTA confirmed PAVFs (Fig. 2b, c).

**Fig. 3:**
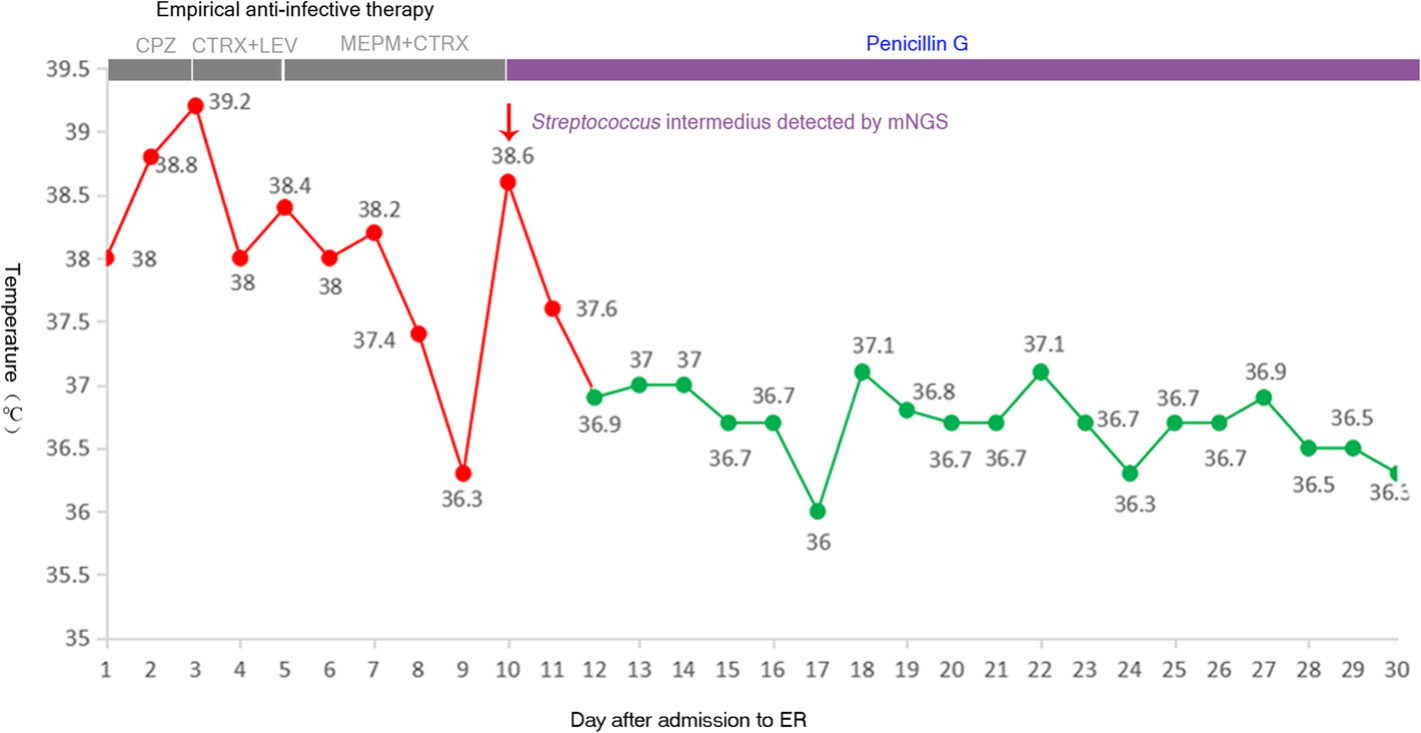
Schematic diagram of the patient’s temperature after admission. On the top of the diagram, various treatment regimen adopted are also listed. As indicated (red arrow), 10 days after admission to ER, when the mNGS results of CSF came back, the patient was given penicillin G as a single therapy to replace previous empirical anti-infection therapy. After that, the patient’s temperature continued normalized without rebound. CPZ, Cefoperazone; CTRX, Ceftriaxone; LEV, Levofloxacin; MEPM, Meropenem

CSF analysis on day 5 after admission showed proteins 1.08 g/L (normal 0.15–0.45 g/L), chlorine 111 mmol/L (normal 120–130 mmol/L), glucose 4.23 mmol/L (normal 2.5–4.5 mmol/L), lactic acid 5.7 mmol/L (normal 1.2–2.1 mmol/L). CSF cytology revealed pleocytosis with WBC count of 960 /mm^3^, NEU% 60, LYM% 36. The patient was received first empirically anti-infection therapy with ceftriaxone (dose 2 g qd) and vancomycin (dose 1000 mg q12h), which was substituted later by meropenem (dose 1 g q12h) as he cannot tolerate the side-effects. This regimen showed only transient effect. The patient’s body temperature sprang back 5 days later when receiving the regimen of meropenem and ceftriaxone. (Fig. 3).

**Fig. 4:**
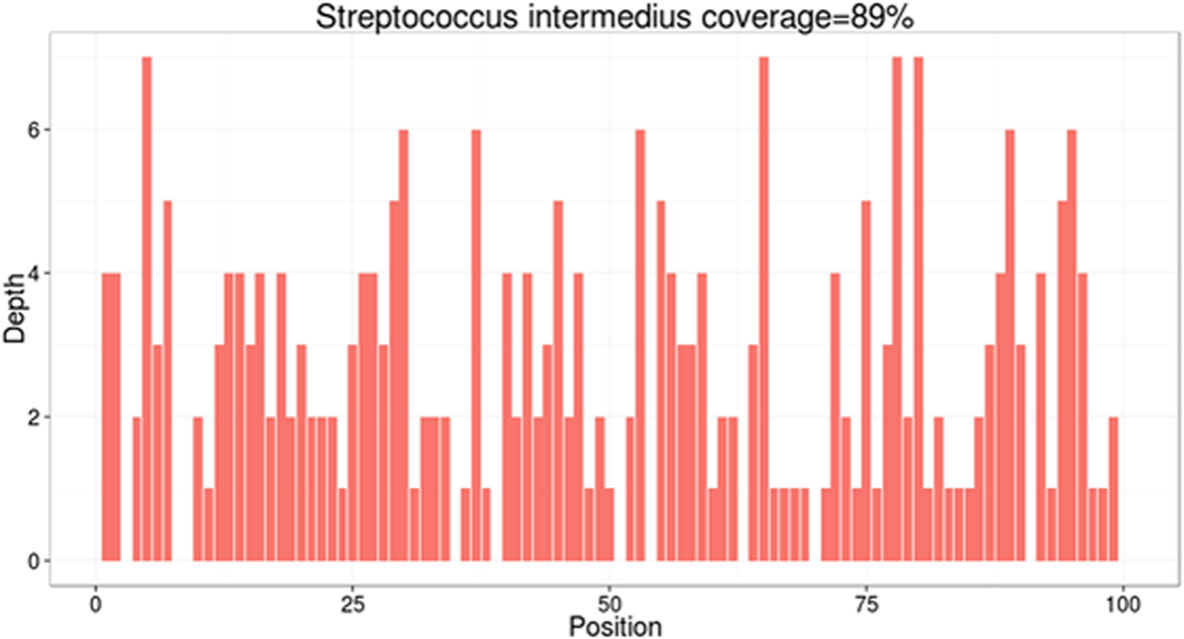
Sequence reads mapped to *S. intermedius* by mNGS data. A total of 261 sequence reads mapped to *S. intermedius* in the reference database, which contains about 1,358,840 pathogen genomes, corresponding to a total coverage of 89%. The X axis represents the position along *S. intermedius* genome. The Y axis represents the number of reads which aligned to the position of the genome

On day 10, the result of metagenomic next-generation sequencing (mNGS) came back, which detected *S. intermedius* as the causative pathogen, while CSF culture was negative. The mNGS was conducted at Oumeng V Medical Laboratory (Guangzhou, China) with following brief protocols. CSF samples were snap-frozen, and stored at − 20 °C until they were delivered to the sequencing center. Total DNA and RNA were extracted from the CSF samples with commercial kit after pretreatment with lysozyme and lyticase, then libraries were constructed and sequenced on Ion Proton platform (Life Technologies, USA). Same protocols were performed for negative control simultaneously. High-quality sequencing data were generated by removing low-quality reads, followed by computational subtraction of human host sequences mapped to the human reference genome (hg19) using Burrows-Wheeler Alignment. The remaining data by removal of low-complexity reads was classified by aligning to the NCBI microbial genome database (ftp://ftp.ncbi.nlm.nih.gov/genomes/) which contains about 1,358,840 pathogen genomes [8]. After analysis, a total of 261 reads were mapped to *S. intermedius* in the reference database. (Fig. 4).

**Fig. 5:**
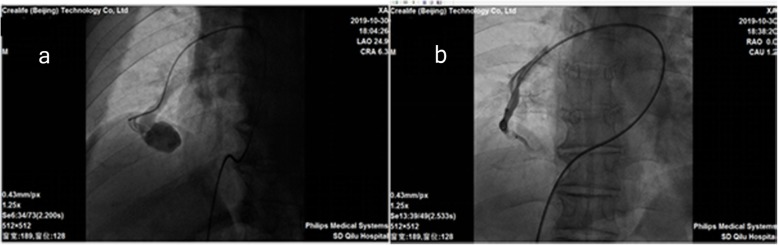
Pulmonary angiography of the right pulmonary artery. **a** pre-embolization image: a 2.5 cm × 2 cm solitary PAVF could be seen in the right pulmonary artery; (**b**) post-embolization image: the feeding artery of the PAVF was successfully occluded by domestic occlusive device

**Fig. 6:**
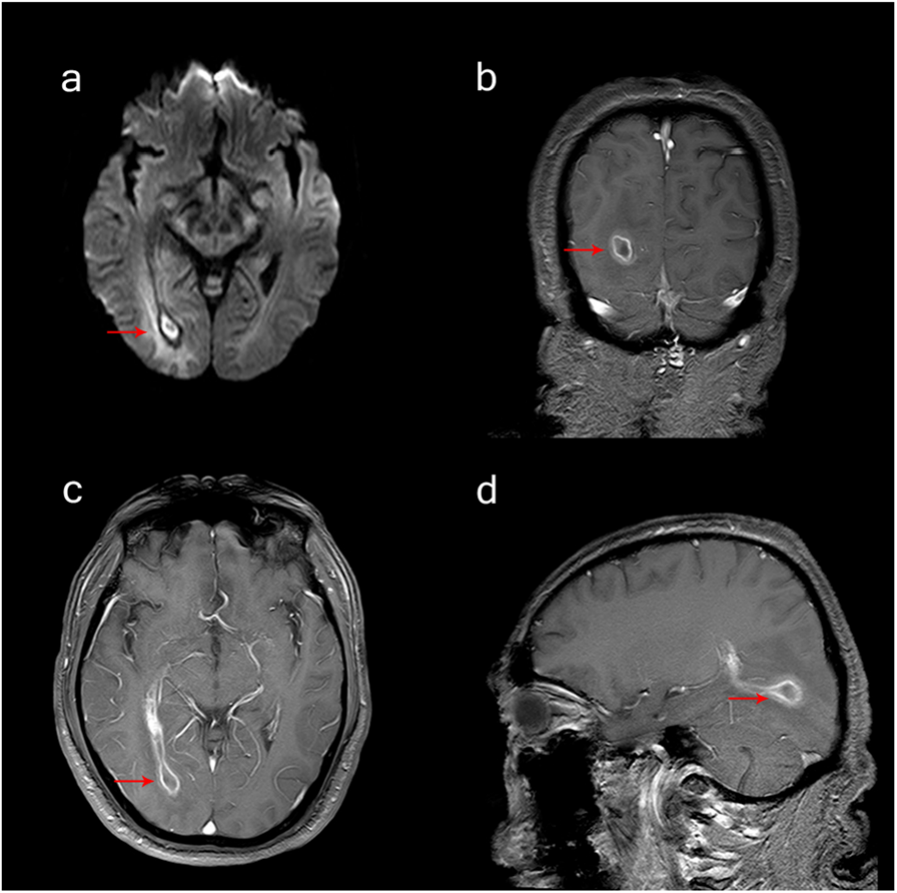
Follow-up brain MRI findings. After 2 weeks Penicillin G treatment, the size of the abscess was significantly reduced: (**a**) DWI and (**b**-**d**) gadolinium enhanced

Based on mNGS result, we discontinued the empirical therapy and chosen penicillin G as the single therapy (dose 6400,000 U q6h). Since then the patient’s temperature turned normal gradually without rebound. (Fig. 3). On day 19, when the patient’s temperature was normalized and the overall condition improved, therapeutic embolization was performed (Fig. 5). On day 24, follow-up brain MRI showed the size of lesion was significantly reduced. (Fig. 6). On day 34, the patient was discharged without any complications.

## Discussion and conclusion

Cerebral abscess is a disease of purulent areas wrapped in the parenchyma of the brain that may be caused by bacteria, fungi, mycobacteria, parasites, etc. [1]. The mortality rate is about 10%, and about 30% of survivors will leave central nervous system symptoms [9]. The predisposing conditions of brain abscess are approximately 40% ~ 50% of an associated contiguous focus of infection, such as sinusitis, subacute or chronic otitis media, systemic infections (eg. infective endocarditis) and right-to-left shunts (such as PAVFs or congenital heart defect) are about 25%, head trauma and neurosurgery occupied 10%, and the rest are cryptogenic [10].

Pulmonary arteriovenous fistula (PAVF), also known as pulmonary arteriovenous malformation (PAVM), is a rare vascular disease that has abnormal communication between the pulmonary artery and vein, leading to a shunt from right to left [11]. Clinical manifestations mainly include cyanosis, exertional dyspnea, and clubbing fingers, while 56% of patients are also asymptomatic [6]*.* It is an important cause of recurrent brain abscess which is rare and easy to neglect in the clinic. Around 80% of them are congenital diseases, in which about 47 ~ 90% of patients are closely related to hereditary hemorrhagic telangiectasis (HHT) [11]. Our patient’s history did not indicate anemia, epistaxis or gastrointestinal bleeding, and additional examinations did not show any evidence of HHT.

Metagenomic next-generation sequencing (mNGS), a promising technique, shows great advantages to identify the causative pathogen from the complicated and serious infections, and to guide precise treatment. It has been reported that the pathogen of the CNS infectious disease remains unknown over 50% with the aiding of traditional diagnostic techniques, such as culture, nucleic acid amplification tests (16S rRNA sequencing) and immunological assays [12]. Compared with traditional diagnostic techniques, the test of mNGS performs more laconic, time-saving and accurate owing to its unbiased analysis of genetic content in a broad range of pathogens that incorporate bacteria, mycobacteria, mycoplasma, chlamydia, fungi, virus and parasite. The pool of detected sequences in the mNGS can match a sequence database of more than 11,300 pathogens in comparison to 16S rRNA sequencing, which can only detect the highly conserved and specific sequences of the bacteria. In clinical practice, more and more physicians have begun to recognize the paramount value of mNGS and apply it to solve infection problems. In this case, the causative pathogen of the abscess, *S. intermedius*, was just identified by mNGS while routine culture results were negative.

*S. intermedius*, a microorganism that usually inhabits in the oral cavity, throat, gastrointestinal flora, and is a part of the normal flora of the human mouth and upper respiratory tract, which is easily detected in suppurative infectious disease such as brain [13] and liver abscesses [14], teeth groove infection [15] and infective endocarditis [16]. *S. intermedius* is highly sensitive to Penicillin G [13], which has broad-spectrum activity, good CNS penetrability and bioavailability. Without confirming pathogens, the empiric anti-infective treatment regimen for brain abscesses is penicillin or cefotaxime or ceftriaxone plus metronidazole, which was effective in most cases [10]. If there is methicillin-resistant *Staphylococcus aureus* (MRSA), vancomycin is recommended [9]. In this case, when empiric combined anti-biotics regimen (ceftriaxone plus vancomycin or meropenem) failed to work, we switched to the single precise therapy of high-dose penicillin G according to the mNGS determined pathogen *S. intermedius*. After treatment, the size of the brain abscess was significantly reduced and no fever recurred. As is recommended that the duration of intravenous treatment for bacterial brain abscess to be at least six weeks for aspirated or conservatively treated abscesses with or without identified pathogen [9], we advised the patient to continue antibiotic treatment for additional 3 weeks after discharge.

For patients with infection-controlled cerebral abscess combined with PAVFs, the pulmonary arteriovenous fistula should be plugged as early as possible to remove the etiology and avoid complications such as recurrent brain abscess and cerebral embolism. Currently, it is considered that endovascular therapy is the preferred treatment for PAVFs, which include spring coil embolization, occlusive device, Onyx drug embolization, venous sac embolization, etc. [11]. Compared with surgery, endovascular therapy has lower complications incidence and shorter hospital stays. In our case, the patient was treated successfully by domestic occlusive device and was discharged 15 days later without any complications.

In conclusion, we described a case of brain abscess associated with PAVFs resulting from infection with *S. intermedius,* which was identified by mNGS while routine blood and CSF culture failed to detect the pathogen. The patient was then treated successfully and economically by Penicillin G. Therefore, it is an alternative way to perform mNGS to identify causative pathogens in patients with brain abscesses especially when the results of traditional culture were negative. Further thoracic CT or pulmonary angiography should also be undertaken to rule out PAVFs as the potential cause of brain abscess if the patient without any known premorbid history.

## Data Availability

All data generated or analyzed during this study are included in this published article.

## Abbreviations

ALT: Alanine transaminase
ANA: Anti-nuclear antibodies
AST: Aspartate aminotransferase
CTA: Computed tomography angiography
CNS: Central nervous system
CSF: Cerebrospinal fluid
ESR: Erythrocyte sedimentation rate
HHT: Hereditary hemorrhagic telangiectasis
mNGS: Metagenomic next-generation sequencing
MIP: Maximum intensity projection
MRI: Magnetic resonance imaging
MRSA: Methicillin-resistant *Staphylococcus aureus*
PAVF: Pulmonary arteriovenous fistula
VRT: Volumetric reproduction technique
